# Combining the Digital, Social and Physical Layer to Create Age-Friendly Cities and Communities

**DOI:** 10.3390/ijerph18010325

**Published:** 2021-01-05

**Authors:** Sonja Pedell, Ann Borda, Alen Keirnan, Nicole Aimers

**Affiliations:** 1 School of Design, Faculty of Health, Arts and Design, Swinburne University of Technology, Hawthorn, VIC 3122, Australia; 2 Centre for Digital Transformation of Health, Faculty of Medicine, Dentistry and Health Sciences, University of Melbourne, Parkville, VIC 3010, Australia; aborda@unimelb.edu.au; 3 Life Without Barriers, Richmond, VIC 3121, Australia; alen.keirnan@lwb.org.au; 4 Summer Foundation, Box Hill, VIC 3128, Australia; nicole.aimers@summerfoundation.org.au

**Keywords:** age-friendly cities, active ageing, social prescribing, wearable technology, digital data layer, age-friendly communities, older adults, citizen science

## Abstract

This qualitative investigation makes suggestions about creating age-friendly cities for older adults focusing on three domains of the World Health Organization (WHO) age-friendly city framework namely “Communication and Information”, “Outdoor Spaces and Buildings” and “Social Participation”. The authors present two case studies, the first one focusing on older adults using activity wearables for health self-management in the neighborhood, and the second one focusing on older adults engaged in social prescribing activities in the community. The authors then reflect on the relationships of the domains and future opportunities for age-friendly cities. These case studies apply a co-design and citizen-based approach focusing within these larger frameworks on emotions, values and motivational goals of older adults. Results suggest how the convergence of the often siloed age-friendly city components based on older adults’ goals and input can lead to better social participation and longer-term health outcomes. The authors propose that the digital, physical and social aspects need to be considered in all domains of age-friendly cities to achieve benefits for older adults. Further work involving older adults in the future shaping of age-friendly neighborhoods and cities, and identifying barriers and opportunities is required.

## 1. Introduction

Physical activity is key for active and healthy ageing, but the main barriers, such as lack of information about appropriate activities or about the environment, prevent older adults from pursuing these activities in a comfortable and safe manner. We propose that access to physical activities through the support of environmental and community infrastructure and digital information is important for older adults to remain independently active as long as possible with opportunities for social participation.

**Figure 1:**
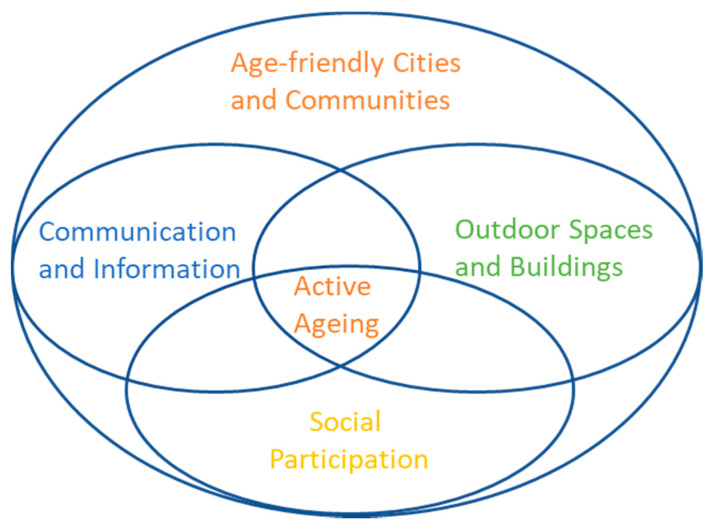
Convergence of age-friendly domains.

Our work looks at active ageing determinants—social and health determinants and their relationship to the physical environment connected through digital technology. The World Health Organization (WHO) launched a world-wide programme for initiating Age-Friendly Cities and Communities in 2007 which includes eight domains or ‘petals’ [1]. We bring together the two domains of “Communication and Information” and “Outdoor Spaces and Buildings” to create a convergent infrastructure that enables “Social Participation” a third domain named in the WHO framework (see Figure 1). We see social participation within a broad context in that actively engaging in the city environment and community spaces can facilitate social encounters and support the choice to socialize. We suggest initiatives are more usefully aligned to older adults when they are enabled to pursue physical activities in their neighbourhood and can take up more easily council services and community offerings tailored to them for active ageing. Such an approach is not easy to achieve as government, health care and research operate in a siloed manner [2] and even the age-friendly domains themselves are looked at separately by councils. However, we suggest collaboration needs to be sustained across different domains and stakeholder groups with a focus on older adults’ needs through a bottom up citizen-based approach. We investigate age-friendly cities for active ageing through the lens of two case studies using qualitative research methods—the first one using activity wearables in the neighbourhood and the second one focusing on social prescribing in the community.

## 2. Case Study 1: Wearing the Smart City: Supporting Older Adults to Exercise by Combining Age-Friendly Environments and Tailored Digital Public Data

In response to the call for more age-friendly cities, this research focused on wearable health technology overcoming some challenges posed by the environment for older adults to be active. Due to the global trend of population ageing, there has been great emphasis placed on ‘healthy ageing’ which is defined as a “lifelong process of optimising opportunities for improving and preserving health and physical, social and mental wellness, independence, quality of life and enhancing successful life-course transitions” [3] (p. 1). In particular, an urgent need has been highlighted to develop strategies to ensure that older people enjoy life in their years and not just extra years in their life [4]. This gives rise to the question of how we can better support the parameters of healthy ageing.

Firstly, there needs to be an understanding of the parameters of digital literacy, without which older adults will experience limitations of the immense potential of the Internet, such as access to public services [5], and other information and communication technology (ICT) such as wearable devices for health self-management [6]. Several studies have revealed the positive effects of internet use and technologies not only on the wellbeing and quality of life of seniors [7], but also in the ability for them to engage in ‘smart’ forms of healthcare [6,8].

Secondly in response to this question, the WHO developed the concept of ‘age-friendly cities’ in order to optimise opportunities for preserving and improving wellness and quality of life [1,9]. As identified by Alley and colleagues [4], age-friendly cities should ideally provide a supportive environment, enabling residents to grow older actively within their families, neighbourhoods, and civic society and present opportunities for their participation in the community.

The convergence of ageing, residing within cities, and age-friendliness, is rapidly producing new modalities to better identify the challenges, such as the notion of ‘urban ageing’, defined as the population of older people living in cities [10]. Such challenges include the creation of inclusive neighbourhoods and the implementation of technology for ageing-in-place and independent living.

According to Kestens and colleagues [11], few studies have considered older adults’ daily mobility to better understand how local urban and social environments may contribute to healthy aging. However, one way in which a better understanding can be gained is through the use of wearable sensors and software applications as they can offer novel means for gathering information on mobility and levels of physical activity [11]. In line with Kestens and colleagues [11], the use of wearable devices in the support and management of independent older adults is becoming more widely advocated [12] and a growing number of seniors are using wearable devices to self-monitor and manage their health [13].

### 2.1. Materials and Method

This case study was part of a larger project building an evidence base focusing on independently living older adults who are using or have used consumer wearable device(s) to self-manage or self-monitor their health [14]. From among the initial cohort of survey respondents, those opting to be interviewed were followed up and comprise this subsequent study. The present study involved a total of eight older adults aged 65 years or older actively using a wearable device(s). The group comprised two male and six female participants, seven of whom fell within the age range 65–69 at the time of the interview, and one in the age range over 80. Among the chronic conditions being ‘managed’ by wearable use included high blood-pressure, arthritis, and obesity. No specific or personal identifying medical information were sought and personal interview data on wearers’ experiences were anonymised. Semi-structured interviews of approximately 60 min were conducted with participants via Skype, phone and/or email. Interview questions were centred around participant experiences and aspirations towards self-management of health using wearable devices.

We applied motivational modelling [15] as the analysis framework. In order to maximise uptake of wearable devices, we propose that more research needs to take place to better understand the functional, quality and emotional goals of older adults when using this technology to maximum benefit within their urban environment. Collectively, these goals form the basis of motivational modelling which not only focuses upon the functionality of solution concepts such as technology (i.e., what it should do) but also considers the social context in which the solution concept is being used. As such, by also modelling the quality goals (i.e., what it should be) and the emotional goals (i.e., how should it feel) of the solution concept, the holistic needs of the end users can be identified which will enable products, systems and services to better support people in their everyday lives [15,16,17]. A thematic analysis of interview transcripts was conducted through the lens of these three thematic frames: The individual user emotions (i.e., how the wearable technology should feel), the qualities of the wearable technology (i.e., what the wearable technology should be) and the functional aspects of the wearable technology (i.e., what the wearable technology should do).

The research reported on in this case study has received ethics approval by the University of Melbourne Human Research Ethics Committee (Ethics ID: 1646991.2).

### 2.2. Findings

#### 2.2.1. Motivational Goals for Active Ageing

A common emotion experienced across all users was a feeling of motivation afforded by the wearable health technologies. This finding aligns with comparable studies [18]. Here, motivation was described by participants as the core driver to use their technology.

While a feeling of motivation was expressed as a core emotion when using wearable health technology, participants described the technology as being a reference point or providing feedback on their participation in exercise which attributed to their motivation. This was seen as the key quality of the wearable device use tied in with motivational behaviour as the following quote shows:

“And then I found that I’d be sitting there till 10:00 or 11:00 in the morning doing some work. And then I realized I’d only done a thousand steps tonight and that would horrify me. So, I’d be out there and make sure […] I did some activity after work and I was much more motivated knowing that I was needing to do more activity.”

The primary functional aspects for which participants described their wearable health technology is to manage their health. Managing one’s health ranged from gaining insights into sleeping behaviours, blood pressure, weight and pain [19].

#### 2.2.2. Wearable Health Technology and the Urban Environment

Several participants directly discussed their interactions with the urban environment. This took various forms. For example, the use of wearable health technology was a motivator for more city walking.

*“If we don’t have much on and we need to go into the city my husband and I will walk into the city so that is 3.5 km. You know I just like to record that.”*

In two cases wearable health technology was a motivator in urban mobility in combination with other interventions, for example.

*“The minimum exercise I have every day is walking to the bus stop and I get off the bus a few stops early and walk up the back streets to the hospital. And that’s even more now because of the changes with the roadworks. Sometimes I’ll get the bus in the morning and walk home at night. I just have to monitor how my hip [is doing] and bail out if it it’s not up to it.”*

In a unique case, one participant who took part in the study from outside Australia (Sweden) became increasingly involved in the use of multiple wearable health devices for health self-management, including the use of a chip implanted in his hand. The latter enabled the participant to engage in a smart city scenario.

*“We use it in our office to open doors and get the printers running and I can use it when I check in to the gym. I can use it when I travel by Swedish railroad. The railroad company can read the chip with their Android phones. The motivational goals in this case are also tempered by emotional ones and perhaps due to the uncommon nature of the adoption of the technology.”* and *“I have it [the chip] in my hand. So that’s sort of a real—you know—discussion starter. Half of the people—no, one third, say “oh that’s terrible”. I would never do that. And it’s really very emotional. It’s not like you know they thought about the pros and cons.”*

This quote demonstrates not only the potential for interacting in more substantial ways with the environment, but also the importance of emotional aspects for uptake.

### 2.3. Discussion

Through three thematic frames of analysis, it was identified that participants wanted to feel motivated when using the technology and, in addition to being a reference point, they wanted the wearable to aid in the management of their health and wellbeing in subtle and controllable ways.

The results show that while the potential design for future wearable health technologies can consider the emotional, quality and functional needs, the environment in which older people experience these attributes is paramount [20]. For example, consider an older person who requires access to public toilets. Their motivation to walk may be present but following through with the action (of walking) may be limited in environments where there are few public conveniences. Likewise, consider an older person who would like to rest under shaded areas on a sunny day. Using their wearable device as a reference point to compare how many steps they did for the day before becomes impractical because again, they are unlikely to go for a walk within environments where there are few shaded areas. Within the present study context, the emotional, quality and functional goals become obsolete when there are environmental barriers within the community, resulting in activity goals unable to be fully achieved. Supporting studies have shown that safe, walkable, and aesthetically designed neighbourhoods, with access to specific destinations and services positively influenced older adults’ physical activity participation [12,21].

It is argued that more research needs to take place to better understand the holistic goals of older adults when using wearable devices within their urban environment and how to overcome barriers posed through the environment. By doing so, insights into framework development for the design of age friendlier cities can be gained.

The present study highlights the opportunities in considering the potential relationship of urban environmental factors within a digital health and urban ageing context. This ‘lens’ has inevitably considered that there are also differing definitional boundaries across individual preferences, health and wellbeing technologies and determinants of health, which require a larger cohort to determine the extent and measures of correlates providing more guidance for age-friendly city strategies. In this study we focused on independently living adults who already were using wearable devices—greater attention also needs to be paid to lower the threshold and increase digital literacy for a wider group of older adults to experience the benefits of wearable devices to be more active in their neighborhoods.

## 3. Case Study 2: Social Prescribing Supporting Social Connectedness in Age-Friendly Communities

Originating in Europe, social prescribing aims for a more holistic health approach to increase social integration while supporting a person’s interests. Through a feasibility study on introducing social prescription in Australia we demonstrate that a bottom-up, neighbourhood-oriented approach is necessary to understand how to overcome barriers to designing a social prescribing service. Social prescribing shows the potential of supporting people to access social activities in the community they are interested in and create new opportunities for combining social and physical activity. We suggest that within the social prescribing concept digital technology can play a key role for people who are vulnerable and easily excluded. People who are digitally and socially connected are safer. Enabling social connections building resilient healthy communities is the responsibility of a holistic health system. Social prescribing is not only relevant for older adults but they have been identified as a main stakeholder group [22].

‘Social prescribing’ is a non-medical referral that links community services with people who are at risk, or experiencing isolation or depression [23]. The person or role prescribing the service can differ from country to country as well as within one country depending on the organisation. Prescribed activities can fall within ‘social’, ‘physical’ or ‘economic’ categories [24] and aim to improve self-care within the community [25]. The literature describes the characteristics of social prescription, stakeholders and models of delivery [23,24,25,26]. Accordingly, the health client journey involves general practitioners working with health clients to determine their level of wellbeing and social interests. In a holistic model of social prescribing, allied health clinicians also play a role in referring health clients to community services. Next, a community connector in collaboration with the health client develops an action plan detailing goals and schedules. Community connectors [25] are people who locate community services for health clients also develop care and well-being plans. A community connector will have strong relationships with umbrella organisations and use their interpersonal skills to help build their health clients’ confidence and independence.

The literature shows promising evidence to the benefits of social prescribing, primarily in the United Kingdom [23,24,25,26] and in Canada [27]. Social prescribing can involve a variety of activities designed to support people with a wide range of social, emotional or practical needs. Services often focus on improving mental health and physical well-being; for example, volunteering, arts activities, group learning, gardening, healthy eating advice and sports [28,29,30]. ‘Nature-based’ social prescribing programs are expanding their reach rather than contracting, despite COVID-19 limitations [31]. In countries such as the UK, U.S., and Canada, there is a grass roots movement among healthcare providers and community and parks services to prescribe physical activity in greenspaces [32], such as Parkrx (https://www.parkrx.org/) managed by the Golden Gate National Parks Conservancy in the San Francisco area and the U.S. National Parks Service. In addition to the physical park spaces and park-based activities around San Francisco which encourage age-friendly activities, there is an openly accessible digital presence as an information hub for “Park Prescriptions” and community resources.

Building spaces also have the potential to draw on social capital and social participation opportunities in a more integrated way, such as museums and cultural venues. Cultural programming is integral to social prescribing referral schemes and they show documented benefits in the involvement of older adults with outcomes of improved psychological wellbeing and social connection [33].

Consequently, social prescribing is located at the crossroads of holistic health, community care and social engagement. Models are still in their infancy and yet to be adopted in Australia but there are strides towards adoption in some form. The COVID-19 predicament in particular has been seen as a timely catalyst for Australia to consider the emerging practice of social prescribing in responding to some of the harmful mental health outcomes of isolation that may not be suitably addressed with conventional medical care [34]. Here, we summarise some key findings on our research for a social prescribing service to be tailored for and piloted in Australia. We focus on the aspects relevant for age-friendly cities and communities.

### 3.1. Materials and Method

The community health provider we collaborated with already offers different portfolios (medical, clinical and community portfolio) of services and hence is uniquely positioned to use existing portfolios as springboard to deliver a social prescribing to their clients. However, the partner was unsure how such a service should be set up and introduced to the community to receive acceptance and longer-term adoption as well as how its success can be determined.

The study recognized the importance of health clients as citizens and co-researchers in the design of holistic healthcare solutions in the community. Co-design, as a participatory design process used in citizen science, collectively involves participants and stakeholders working together through active participation from the design stage of research to the interpretation of research results and to their transformation into concrete actions. This process makes full use of participants’ knowledge, resources and contributions, to achieve better outcomes or improved efficiency in health research or service design (for example [35,36]).

We wanted to ensure representation from different departments, and using the literature about social prescribing highlighted departments and/or job functions that were previously described in the different UK equivalents. These included intake officers, community connectors, physicians and specialist therapist groups. With our selection criteria, we sent targeted emails to these groups for the purpose of recruitment for the first staff workshop. Staff in relevant service areas identified in this workshop were invited to the second workshop.

During the staff workshops, there was a thread describing who in the community were likely to be service users. These included people who were new parents, people who are migrating from one country to another, social technology-dependent people, night shift workers and older adults. These themes formed the basis of our selection criteria. Leveraging the Future Self and Design Living Lab community pool, six key community members who fit within these themes were contacted and asked to participate (age range in their twenties to nineties). As a result, a new parent (1), recently migrated (2), and older adults (3) participated in the workshop. More details on the recruitment process can be found in [22].

Michael Schrage [37] notes: “Innovation is not innovators innovating but customers adopting” (p. 91). Innovation should only be measured by the value it creates to people’s lives. Hence, what often is missing is an approach that spans from designing to impact measurements of holistic health and community services that include the voice of the users. This is a key motivator of the present study and in the selection of methodologies. Also, interventions, as well as decision-making, are more effective when the target group is engaged in an equitable partnership [35]. The co-design process took place over the course of several iterative engagements through interviews and workshops.

Aims (with stakeholders and methods in brackets) were to:

1. Explore resources, enablers and barriers for a social prescribing service in the community (interviews and focus group with staff);
2. Explore emotions, values, qualities and goals of a social prescribing service with potential clients (focus group with community members);
3. Co-design a conceptual scenario-based service model with key stakeholders (focus group with staff);
4. Use citizen science as a model to maintain participatory approaches to shape social prescribing services as part of a bigger learning system (outlook on evaluation and sustainability)

We conducted seven interviews with the organisation’s staff from different service areas to better understand the whole existing ecosystem. Building on the interviews and knowledge of the ecosystem gained two co-design workshops with health practitioners and one client workshop were facilitated to understand the goals of the respective stakeholder groups and the user journey throughout the social prescribing service. All three workshops were designed for the results to cascade into the next, ensuring the co-design process was open and flexible. The research for this case study has received ethics approval by the Swinburne Human Research Ethics Committee (ID 2016/144).

### 3.2. Findings

#### 3.2.1. Barriers within the Community Health Provider’s Ecosystem

The data collected with staff confirmed three portfolios which should ‘ideally’ refer services to each other and barriers preventing communication and client transfers where revealed. The rapid growth of the organisation was pointed out as a potential barrier to communication among the three portfolios creating silos. Established and one-directional pathways within the organisation prevented the clients from moving from one portfolio to another. In particular, the lack of mental health awareness was discussed as a whole of organisation barrier which also would affect the referral onward to a social prescription service in the community. Other barriers included time poor staff, broken pathways and channels, but also an assumed limited motivation and interest of clients.

The staff interviews reinforced the literature findings that social prescribing is not based on a traditional medical model, but needs to be tied in with the community structures:

*“Social prescribing is looking at someone as a whole. It’s a holistic approach to talking about someone’s care”* and *“Ways they can prescribe things for them to do socially that will assist them for their health rather than just drugs they can take.”*

Importantly its success is determined on how people want to feel and engage:

“…ways that people can help themselves to improve their wellbeing or engage in an activity to help them benefit their mood.”

Consequently, the involvement of future service recipients is key to the social prescription concept. A client workshop with community members was organised to address this. A two-hour workshop with six potential clients revealed important insights about emotions, goals, tangible aspects of social prescription and, importantly, underlying values.

#### 3.2.2. Values and Goals Expected from a Social Prescribing Service (Community Member Workshop)

**Figure 2:**
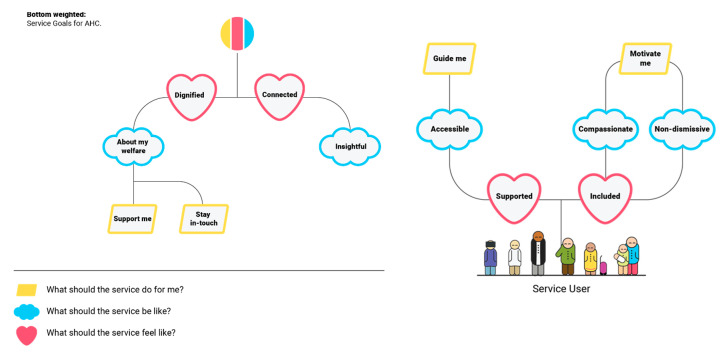
Goals (do/be/feel) that a social prescribing service should do according to the community.

The workshop with clients produced two main outcomes. The first outcome is describing the different values of a social prescription. It shows the different characteristics and values of a social prescription that clients would like to have embedded into their experience. The second outcome, a goal model, accompanies the values demonstrating the preferred emotions, qualities and functions that a health client would like to interact with during their social prescription service (see Figure 2).

Four values were deemed necessary if clients were to engage with a social prescription offering by the community health provider. A sense of connection (i) to the greater community was described as integral to the social prescribing journey. This might be a simple referral to a wider network of activities outside the organisation after a period of time. Clients also wanted to feel safe and comfortable (ii) with their clinicians and not stigmatised. This also included to be able to determine their own course of action.

The clinician should feel trustworthy (iii) that the clients could feel confident in their abilities e.g., a sense of knowing that the health provider is aware of mental health illness and how to diagnose and treat such illnesses were important. Finally, clients wanted a real sense of having a tailored approach (iv) to their social prescription. This included that their interests should be known. Upholding these values were associated emotions, qualities and functions as shown in Figure 2. Across all goals it is apparent how digital technology can play a key role in a successful community service achieving qualities such as being accessible in also providing information online and non-dismissive in taking on board needs and feedback over time. Functional goals such as the community service provider staying in touch that service recipients are feeling connected and supported could also be facilitated by technology.

A final co-design workshop with staff confirmed the values and goals collected with the service recipients. This led to a service concept based on the goals and values of the community.

### 3.3. Discussion

We framed the involvement of the multiple stakeholder groups as one informed by citizen science to understand how to overcome organisational and environmental barriers. The result is a concept proposal that suggests service pathways for a social prescription based on the healthcare providers’ and their stakeholders’ values and needs. Older adults were identified as key stakeholder group. Our approach can inform other health-related services in the community giving older adults a stronger voice in the design, implementation and maintenance of wellbeing services that extend the traditional medical model of health and lead to more age-friendly cities.

The aim of the program logic (part of the service blueprint) was to capture whether the inputs, activities and outputs will lead to the desired outcomes according to the results from the client and staff consultation. To evaluate the outcomes of a social prescription trial service, and refine the service to a sustainable model, meaningful data, capturing the successes, failures, and positive or negative journey experiences for all stakeholders are necessary. Values and client goals become key to the evaluation over time.

A learning healthcare system (LHS) is broadly defined as: “…one that is designed to generate and apply the best evidence for the collaborative healthcare choices of each patient and provider; to drive the process of discovery as a natural outgrowth of patient care; and to ensure innovation, quality, safety, and value in health care” [38].

Since this original definition, there has been increasing recognition of the need to engage with various stakeholders including patients, participants, health care providers, and policy-makers among others to understand how to drive a sustainable LHS [39]. The bottom-up approach of citizen science involving diverse stakeholders and localised problem-solving is an effective way to translate knowledge to a broader audience and to support iterative evaluation processes within an LHS [40]. A post-implementation stage of evaluation, for example, offers opportunities to engage citizens to monitor problems, and to facilitate an open exchange of various perspectives, and thereby improve mutual understanding without some of the limitations of formal research methods.

In this way, citizen science participation can be more open among other methodological approaches in that the citizens do not always need to be pre-selected by researchers or healthcare providers [41,42]. This allows for an agile model that focuses on evolving community needs rather than producing generalisable knowledge and which closely aligns with the notion of an LHS [39,43].

## 4. Discussion on Converging Perspectives

Taken together the two case studies illustrate a convergence of the three domains or petals of age-friendly cities, “Communication and Information” and “Outdoor spaces and buildings”, leading to better “Social Participation” which are collectively undergoing a dramatic digital transformation as a result of the challenges created by COVID-19 [44,45]. These challenges present a unique opportunity to understand the future ways wearable technologies and communication technologies that can be integrated into digitally enabled age-friendly cities and community context and support the immediate social needs of older adults.

### 4.1. Communication and Information

Going forward, there is an urgent need to enable equitable access to the Internet, and digital enabling technologies, such as wearable technologies, especially if these are socially or medically prescribed [46]. The recently published Topol Review [47] notes that technology has the potential to worsen health inequality if not used correctly. In a move towards smarter cities and communities, in particular, there is a universal recognition that vulnerable people often are those who are not digitally literate [48], and thereby become excluded from accessing health interventions and community services increasing the digital divide in particular for older adults. Digital literacy requires the embedding of digital skills and accessible training, for instance, in a dedicated social prescribing program which can be available to intergenerational communities [49].

With this potential, citizens, such as older adults, can leverage smart cities with its digital technologies and Internet of Things (IoT)-enabled infrastructure to provide actionable insights that help improve their health and well-being [8]. Older adults, for instance, can use ICT to gather and share information about themselves and the environment that surrounds them. Projects like the European funded PULSE (Participatory Urban Living for Sustainable Environments) is developing a public health observatory, with the participation of intergenerational citizen scientists using wearable devices [50] across seven cities: Paris, Singapore, Birmingham, Barcelona, New York, Pavia, and Keelung.

### 4.2. Outdoor Spaces and Buildings

Our ability to connect spaces with people and healthy communication is key in this direction. Social prescribing, for instance, offers a capability to join up aspects of this—especially if a programme can integrate both physical and digital access—e.g., digital apps and wearables supporting older adults in their interactions in the built environment or local neighbourhood, whether for increased exercise, lower level mental health issues such as mild anxiety, and social isolation through chat applications and smart walking guides, for example, den Haan et al. [51].

In line with the ‘age friendly’ cities framework in which outdoor spaces and buildings are identified as a domain of city life which can assist with active and healthy ageing, it is argued that there is a further need for public infrastructure data sets. Specifically, if a digital city layer showing, for example where facilities such as public toilets, water fountains and shaded rest stops were located on exercise routes in communities, it is possible that this would support older people in achieving their exercise goals and ultimately, optimise opportunities for preserving and improving wellness and quality of life. This could be available as an app which would allow older people to download directly to their smart wearable device and customise the information, depending on where they are located and their preferences to show the information needed [11,52]. Given the continual advancements in wearable technology, the ability to tailor this information for each older person and integrate this information in an easy to understand way on their smart device is becoming achievable [53]. However, this means that the digital information layer about the environment needs to exist along with connectivity to the wearable device. Critically, a framework is required to be in place in which the individual and community can responsibly share the data, as well as govern it [6].

### 4.3. Social Participation

Studies show that sustained community engagement requires creative approaches to promote the wellbeing and social involvement of older adults and vulnerable individuals [54]. The co-design of community apps and the use of digital storytelling are among the effective ways of supporting age-friendly cities and communities [55]. An acknowledged gap is how technologies can be used and deployed across different communities and how these can be improved, adopted and innovated by communities of older adults [21]. There are untapped areas of technology adoption, for instance, such as gamification which may lend itself to support different social interactions in outdoors and building spaces, beyond what might be assumed as stereotypically a younger generational platform [56].

Partnerships of researchers and community agencies are integral in collaborating directly with communities so that social solutions can move beyond the generic and placeless, and become embedded into specific locally relevant programming to better connect the individual and community to a place in age-friendly ways.

Here we argue that in the context of age-friendly cities, there is a need to connect to the smart city discussion by breaking up silos of technology, the environment and human-centred design. In putting forward the goals of older adults, we acknowledge the existence of different needs and how they can be accomplished through knowledge about the environment and through forms of social participation. Given that wearable devices are increasingly being used by older adults, it is important to take the next step to connect the environment, socio-technological considerations, and the user in order to create a holistic system that supports the quality of life of an ageing person.

### 4.4. Limitations

This present study was limited to three domains of the Age-Friendly Cities and Communities Framework and the relationships across these. It was not intended to provide a comprehensive approach to the topic—but rather an exploratory one that highlights the opportunities in considering the potential relationships across the environment and ICT factors within a digital health and age-friendly context. This lens has inevitably taken into account the outcomes of the two case studies that highlight differing definitional boundaries across individual and community preferences, technologies and determinants of health. For instance, there were potential differences in the adoption of wearable technologies and literacies in health self-management. Subsequent studies will require a larger sample cohort to determine the extent and measurements of correlates. Visual analyses would further provide a means to examine social determinants of health, urban and geolocative features in more detail in order for these to translate into concrete recommendations for age-friendliness city and community strategies.

## 5. Conclusions

This paper has focused on three age-friendly city components and the benefits of their convergence to potentially help activate changes that can improve older peoples’ health and support their social participation in neighbourhoods and communities. Through case study examples, the significance of place in the lives of older people and how they can participate underpin the importance of their surrounding environments as sources of meaning and self-identity.

In particular, support for active participation through digitally-enabled platforms can lead to sustained independence and reduced risk of isolation, for example, through the availability of appropriate communication and information to help maintain relationships and networks, as well as providing safer access to services and amenities. Thus, the reasons for such convergence are not only about creating an age-friendly environment, but they are also necessarily linked to increasing the years of quality of life.

Notwithstanding this, there needs to be much further work in building up real-world examples of interventions involving older adults in the future that shape age-friendly neighbourhoods and cities, as well as in identifying barriers to and opportunities from their participation. This can be in the form of the application of assistive technology in the community to navigate local environments as time spent outdoors or to reach amenities, or other supporting forms of social interaction and the development of social networks with consequential benefits for physical and mental health. The need for places where citizens regardless of their age will feel secure and capable is a significant challenge not least in terms of the range of experiences by different groups and their social determinants of health—but this is the start of the basis for re-designing age-friendly and smart communities directly and collaboratively with those affected communities in order to achieve such a goal.

## Data Availability

The data presented in this study are available on request from the corresponding author. The data are not publicly available due to ethical approvals at the time.

## References

[1] World Health Organization (2007). Global Age-Friendly Cities: A Guide; Report. https://www.who.int/ageing/publications/Global_age_friendly_cities_Guide_English.pdf.

[2] Greenfield E.A., Oberlink M., Scharlach A.E., Neal M.B., Stafford P.B. (2015). Age-friendly community initiatives: Conceptual issues and key questions. Gerontologist.

[3] Health Canada (2001). Workshop on Healthy Aging. http://publications.gc.ca/collections/Collection/H39-612-2002-1E.pdf.

[4] Alley D., Liebig P., Pynoos J., Banerjee T., Choi I.H. (2007). Creating elder-friendly communities: Preparations for an aging society. J. Gerontol. Soc. Work.

[5] Caruso L. (2018). Digital innovation and the fourth industrial revolution: Epochal social changes?. AI Soc..

[6] Cosco T.D., Firth J., Vahia I., Sixsmith A., Torous J. (2019). Mobilizing mHealth data collection in older adults: Challenges and opportunities. JMIR Aging.

[7] Gustafson D.H., McTavish F., Gustafson D.H., Mahoney J.E., Johnson R.A., Lee J.D., Quanbeck A., Atwood A.K., Isham A., Veeramani R. (2015). The effect of an information and communication technology (ICT) on older adults’ quality of life: Study protocol for a randomized control trial. Trials.

[8] Cook D.J., Duncan G., Sprint G., Fritz R.L. (2018). Using Smart City Technology to Make Healthcare Smarter. Proc. IEEE.

[9] WHO (2018). The Global Network for Age-friendly Cities and Communities: Looking Back over the Last Decade, Looking forward to the Next. https://www.who.int/ageing/publications/gnafcc-report-2018/en/.

[10] van Hoof J., Kazak J.K., Perek-Białas J.M., Peek S.T.M. (2018). The challenges of urban ageing: Making cities age-friendly in Europe. Int. J. Environ. Res. Public Health.

[11] Kestens Y., Chaix B., Gerber P., Desprès M., Gauvin L., Klein O., Klein S., Köppen B., Lord S., Naud A. (2016). Understanding the role of contrasting urban contexts in healthy aging: An international cohort study using wearable sensor devices (the CURHA study protocol). BMC Geriatr..

[12] Barnett D.W., Barnett A., Nathan A., Van Cauwenberg J., Cerin E. (2017). Built environmental correlates of older adults’ total physical activity and walking: A systematic review and meta-analysis. Int. J. Behav. Nutr. Phys. Act..

[13] McMahon S.K., Lewis B., Oakes M., Guan W., Wyman J.F., Rothman A.J. (2016). Older adults’ experiences using a commercially available monitor to self-track their physical activity. JMIR mHealth uHealth.

[14] Borda A., Gilbert C., Gray K., Prabhu D., Van den Berg M., Maeder A. (2018). Consumer wearable information and health self management by older adults. Studies in Health Technology and Informatics.

[15] Sterling L.S., Taveter K. (2018). The Art of Agent-Oriented Modeling.

[16] Miller T., Pedell S., Sterling L., Vetere F., Howard S. (2012). Understanding socially oriented roles and goals through motivational modelling. J. Syst. Softw..

[17] Marshall J. (2014). Agent-Based Modelling of Emotional Goals in Digital Media Design Projects. Int. J. People-Oriented Program..

[18] Kononova A., Li L., Kamp K., Bowen M., Rikard R.V., Cotten S., Peng W. (2019). The use of wearable activity trackers among older adults: Focus group study of tracker perceptions, motivators, and barriers in the maintenance stage of behavior change. J. Med. Internet Res..

[19] Kim B.Y.B., Lee J. (2017). Smart devices for older adults managing chronic disease: A scoping review. JMIR mHealth uHealth.

[20] Ehn M., Johansson A.C., Revenäs Å. (2019). Technology-based motivation support for seniors’ physical activity—A qualitative study on seniors’ and health care professionals’ views. Int. J. Environ. Res. Public Health.

[21] Tuckett A.G., Freeman A., Hetherington S., Gardiner P.A., King A.C. (2018). Older adults using our voice citizen science to create change in their neighborhood environment. Int. J. Environ. Res. Public Health.

[22] Pedell S., Borda A., Keirnan A. (2020). Social Prescribing in Australia: How the Bottom-up Model of Citizen Science Can Facilitate Stakeholder Engagement in Health Service Design. Proceedings of DLLD 2020.

[23] Carnes D., Sohanpal R., Frostick C., Hull S., Mathur R., Netuveli G., Tong J., Hutt P., Bertotti M. (2017). The impact of a social prescribing service on patients in primary care: A mixed methods evaluation. BMC Health Serv. Res..

[24] Woodall J., Trigwell J., Bunyan A.M., Raine G., Eaton V., Davis J., Hancock L., Cunningham M., Wilkinson S. (2018). Understanding the effectiveness and mechanisms of a social prescribing service: A mixed method analysis. BMC Health Serv. Res..

[25] Moffatt S., Steer M., Lawson S., Penn L., O’Brien N. (2017). Link Worker social prescribing to improve health and well-being for people with long-term conditions: Qualitative study of service user perceptions. BMJ Open.

[26] Kimberlee R. (2015). What Is Social Prescribing?. Adv. Soc. Sci. Res. J..

[27] Mulligan K., Hsiung S., Bhatti S., Rehel J., Rayner J. (2020). Rx: Community Social Prescribing in Ontario. Final Report.

[28] Polley M., Fleming J., Anfilogoff T., Carpenter A. (2017). Making Sense of Social Prescribing.

[29] Husk K., Blockley K., Lovell R., Bethel A., Lang I., Byng R., Garside R. (2020). What approaches to social prescribing work, for whom, and in what circumstances? A realist review. Health Soc. Care Community.

[30] Buck D., Ewbank L. What Is Social Prescribing?. https://www.kingsfund.org.uk/publications/social-prescribing.

[31] Kondo M.C., Oyekanmi K.O., Gibson A., South E.C., Bocarro J., Hipp J.A. (2020). Nature prescriptions for health: A review of evidence and research opportunities. Int. J. Environ. Res. Public Health.

[32] Leavell M.A., Leiferman J.A., Gascon M., Braddick F., Gonzalez J.C., Litt J.S. (2019). Nature-Based Social Prescribing in Urban Settings to Improve Social Connectedness and Mental Well-being: A Review. Curr. Environ. Health Rep..

[33] Thomson L.J., Lockyer B., Camic P.M., Chatterjee H.J. (2018). Effects of a museum-based social prescription intervention on quantitative measures of psychological wellbeing in older adults. Perspect. Public Health.

[34] Wells L. (2020). ‘Iso’—A Spur to Think about Social Prescribing. Croakey.

[35] Den Broeder L. (2017). Citizen Science for Health in All Policies. Ph.D. Thesis.

[36] Wiggins A., Wilbanks J. (2019). The Rise of Citizen Science in Health and Biomedical Research. Am. J. Bioeth..

[37] (2008). Ubiquity staff An Interview With Michael Schrage On Ubiquity. Ubiquity.

[38] Olsen L., Aisner D., McGinnis J.M. (2007). The learning healthcare system: Workshop summary. IOM Roundtable Evid. Based Med..

[39] Platt J.E., Raj M., Wienroth M. (2020). An analysis of the learning health system in its first decade in practice: Scoping review. J. Med. Internet Res..

[40] Van Brussel S., Huyse H. (2019). Citizen science on speed? Realising the triple objective of scientific rigour, policy influence and deep citizen engagement in a large-scale citizen science project on ambient air quality in Antwerp. J. Environ. Plan. Manag..

[41] Borda A., Gray K., Downie L. (2019). Citizen Science Models in Health Research: An Australian Commentary. Online J. Public Health Inform..

[42] Sauermann H., Vohland K., Antoniou V., Balázs B., Göbel C., Karatzas K., Mooney P., Perelló J., Ponti M., Samson R. Citizen Science and Sustainability Transitions. https://ssrn.com/abstract=3511088.

[43] Petersen C., Austin R.R., Backonja U., Campos H., Chung A.E., Hekler E.B., Hsueh P.-Y.S., Kim K.K., Pho A., Salmi L. (2020). Citizen science to further precision medicine: From vision to implementation. JAMIA Open.

[44] Capolongo S., Rebecchi A., Buffoli M., Appolloni L., Signorelli C., Fara G.M., D’Alessandro D. (2020). COVID-19 and Cities: From Urban Health strategies to the pandemic challenge. A Decalogue of Public Health opportunities. Acta Biomed..

[45] Ogden J. (2020). Social Prescribing In A Time Of Covid-19 And Social Isolation. Prog. Neurol. Psychiatry.

[46] Jungmann S., Mistry P., Conibear T., Gray M., Jani A. (2020). Using technology-enabled social prescriptions to disrupt healthcare. J. R. Soc. Med..

[47] Topol E. Preparing the Healthcare Workforce to Deliver the Digital Future The Topol Review. An Independent Report on Behalf of the Secretary of State for Health and Social Care. https://topol.hee.nhs.uk.

[48] OECD Smart Cities and Inclusive Growth. https://www.oecd.org/cfe/cities/OECD_Policy_Paper_Smart_Cities_and_Inclusive_Growth.pdf.

[49] Good Things Foundation (GTF) Socially Prescribing Digital Skills: A How to Guide for Digital Inclusion in Health. https://www.onlinecentresnetwork.org/sites/default/files/how_to_socially_prescribing_digital_skills_in_health_v2.pdf.

[50] Ottaviano M., Beltrán-Jaunsarás M.E., Teriús-Padrón J.G., García-Betances R.I., González-Martínez S., Cea G., Vera C., Cabrera-Umpiérrez M.F., Waldmeyer M.T.A. (2019). Empowering citizens through perceptual sensing of urban environmental and health data following a participative citizen science approach. Sensors.

[51] den Haan M.C., Brankaert R.G.A., Lu Y., Christer K., Craig C., Wolstenholme D. (2018). What moves you? Designing a walking app for and with older adults. Proceedings of the Design4Health, Sheffield Hallam University, Sheffield, UK, 4–6 September 2018.

[52] Cuignet T., Perchoux C., Caruso G., Klein O., Klein S., Chaix B., Kestens Y., Gerber P. (2020). Mobility among older adults: Deconstructing the effects of motility and movement on wellbeing. Urban Stud..

[53] Baraković S., Husić J.B., Van Hoof J., Krejcar O., Maresova P., Akhtar Z., Melero F.J. (2020). Quality of life framework for personalised ageing: A systematic review of ICT solutions. Int. J. Environ. Res. Public Health.

[54] Cinderby S., Cambridge H., Attuyer K., Bevan M., Croucher K., Gilroy R., Swallow D. (2018). Co-designing Urban Living Solutions to Improve Older People’s Mobility and Well-Being. J. Urban Health.

[55] Marston H., Van Hoof J. (2019). Who Doesn’t Think about Technology When Designing Urban Environments for Older People?. Int. J. Environ. Res. Public Health.

[56] Marston H.R., Kroll M., Fink D., Poveda R., Gschwind Y.J., Marston H., Freeman S., Musselwhite C. (2017). Digital Game Technology And Older Adults. Mobile e-Health. Human.

